# Ancient mitochondrial DNA connects house mice in the British Isles to trade across Europe over three millennia

**DOI:** 10.1186/s12862-021-01746-4

**Published:** 2021-01-23

**Authors:** Oxala García-Rodríguez, Emilie A. Hardouin, Ellen Hambleton, Jonathan Monteith, Clare Randall, Martin B. Richards, Ceiridwen J. Edwards, John R. Stewart

**Affiliations:** 1 Faculty of Science and Technology, Bournemouth University, Christchurch House, Talbot Campus, Poole, BH12 5BB Dorset UK; 2 Department of Biological and Geographical Sciences, School of Applied Sciences, University of Huddersfield, Huddersfield, UK

**Keywords:** Ancient DNA, Phylogeography, Mitochondrial DNA, House mouse, *Mus musculus*, Britain

## Abstract

**Background:**

The earliest records in Britain for the western European house mouse (*Mus musculus domesticus*) date from the Late Bronze Age. The arrival of this commensal species in Britain is thought to be related to human transport and trade with continental Europe. In order to study this arrival, we collected a total of 16 ancient mouse mandibulae from four early British archaeological sites, ranging from the Late Bronze Age to the Roman period.

**Results:**

From these, we obtained ancient mitochondrial DNA (mtDNA) house mouse sequences from eight house mice from two of the sites dating from the Late Bronze to Middle Iron Age. We also obtained five ancient mtDNA wood mouse (*Apodemus* spp.) sequences from all four sites. The ancient house mouse sequences found in this study were from haplogroups E (N = 6) and D (N = 2). Modern British house mouse mtDNA sequences are primarily characterised by haplogroups E and F and, much less commonly, haplogroup D.

**Conclusions:**

The presence of haplogroups D and E in our samples and the dating of the archaeological sites provide evidence of an early house mouse colonisation that may relate to Late Bronze Age/Iron Age trade and/or human expansion. Our results confirm the hypothesis, based on zooarchaeological evidence and modern mtDNA predictions, that house mice, with haplogroups D and E, were established in Britain by the Iron Age and, in the case of haplogroup E, possibly as early as the Late Bronze Age.

## Background

The western European house mouse (*Mus musculus domesticus*) is today a widely distributed commensal species that is closely associated with human settlements. Although likely a commensal since Neolithic times in the Near East, with rare evidence for its presence in Europe during the Bronze Age [1, 2], it has been argued, from zooarchaeological analyses, that it probably did not spread widely into western Mediterranean and northern Europe until the first millennium BC, during the Late Bronze Age or Iron Age [3]. The earliest records in Britain have been thought to date from the Late Bronze Age [4–7]. However, as these are not from securely dated stratigraphic contexts [8], it is possible that the house mouse did not arrive and become fully established Britain until the emergence of the denser human settlements of the Iron Age [3]. This may have initially taken place in southern England [9, 10], where the presence of structures to store cereal grain, which represent an ideal niche for house mice, would have helped their introduction, although cereals were available from the Neolithic [11]. While identification of their physical remains is not always straightforward, genetic evidence can give clearer results.

Within the last 3000 years, the British Isles have experienced multiple waves of human immigration and a long history of contact, trade and exchange with continental Europe. Due to the close relationship between the two species, demographic changes in human populations are likely paralleled by similar changes in house mouse numbers; indeed, the house mouse niche has largely been shaped by humans [12]. Indeed, phylogeographic studies have shown that historical human movements have impacted on current house mouse population patterns [7, 12–15]. However, to date, the founding populations and colonisation routes have been identified based on modern phylogeography alone. Ancient DNA (aDNA) analyses of early house mice from Great Britain can test these conclusions and help us to understand the origins of the earliest house mouse populations in the British Isles.

In this study, we aim to characterise the first colonisation of the western European house mouse in Great Britain, and the possible route that these first arrivals followed, based on ancient DNA. To this end, we analysed mitochondrial DNA (mtDNA) control-region sequences of some of the rare ancient house mouse remains from four archaeological sites in southern England, dating from the Late Bronze Age to the Middle Iron Age and Roman period, and compared these with published modern European mtDNA control-region data.

## Methods

### Archaeological samples

The archaeological record of small rodents is adversely affected by their small size, and sieving of sediment using a maximum mesh of 2 mm is necessary for their recovery [11, 16]. The small size of the bones also makes them problematic for radiocarbon dating, as a single bone will rarely yield enough collagen for direct analysis. Therefore, the dating of mouse material often requires dating by context and association with artefacts and, consequently, lacks reliability and precision.

We collected a total of 16 ancient mouse mandibulae from four British archaeological sites, ranging from the Late Bronze Age to the Roman period (see Additional File 1: Fig. S1; Table S1)—Potterne, Wiltshire (*n* = 7); Battlesbury Bowl, Wiltshire (*n* = 5); North West Farm, Dorset (*n* = 2); and Druce Farm Roman Villa, Dorset (*n* = 2).

The site of Potterne, near Devizes, Wiltshire, was excavated by the commercial archaeological unit Wessex Archaeology between 1982 and 1984, and comprises an extensive accumulation of dark anthropogenic soil deposits, up to 2 m deep in places, covering an area of 3.5 ha. The ‘midden-like’ deposits were rich in artefacts and biological remains, resulting from the accumulation of manure and refuse from stock keeping, and the repeated dumping and trampling of waste from human occupation and activities on and around the site over a 500-year period. Pottery typology, and radiocarbon dating of charcoal from different levels within the deposit and other cut features, suggest a date of 1200–600 BC, encompassing the Late Bronze Age into the very Early Iron Age period [6]. In addition to a large animal bone assemblage dominated by domestic mammals, small mammal remains were recovered, mainly from sieved environmental samples, and house mouse remains were identified from every level [17]. Although it was not possible to obtain direct radiocarbon dates from the mice themselves to confirm the Late Bronze Age date, a radiocarbon date of 1,460–990 cal. BC (2ơ; lab number HAR-8938) was obtained from charcoal that came from the same posthole as mouse mandible OG06 [6]. The assumption is that all seven mouse samples (OG01–OG07) are contemporaneous with the associated archaeological materials of the Late Bronze Age (*c*. 1200–600 cal. BC) and date from the same layers and contexts; although we note that Locker [17] does caution that some small mammal remains may have filtered down the deposit from higher levels.

The later prehistoric site of Battlesbury Bowl lies along a narrow chalk ridge immediately to the north of Battlesbury Camp, an Iron Age hillfort near Warminster, Wiltshire. Excavations by Wessex Archaeology in 1999 revealed features of Late Bronze Age to Middle Iron Age dates (based on ceramic style), including ditches, post holes, and almost 200 pits [18]. The faunal assemblage is one of the largest collections of Early to Middle Iron Age faunal material from Great Britain. Hambleton and Maltby [19] report the presence of both house mouse and wood mouse in both the hand-recovered assemblage and the environmental sieved samples. The mouse mandibulae included in this study came from pit fills (OG08, OG09, OG11, OG12) and a posthole (OG10), all of which were assigned Early to Middle Iron Age dates. Radiocarbon dating of a pig humerus, from the same context as mouse mandible OG11, provided a date of 420–100 cal. BC (2ơ; lab number NZ-13634) [18].

The site at North West Farm is just outside the village of Winterborne Kingston to the north of Bere Regis, Dorset. The archaeology represents multiple phases spanning Bronze Age to Roman periods. It forms part of a program of archaeological fieldwork, the Durotriges Project, designed to investigate native and Romano-British settlement across Dorset, focusing specifically on the archaeologically distinct Iron Age Durotriges tribe. Two mouse remains (OG13 and OG14) were recovered from a chalk deposit (340) within one of three large storage pits in Trench H of the 2017 fieldwork season and, based on preliminary pottery attributions and the form of the pits, these may date to the Bronze Age.

Druce Farm Villa, Puddletown, Dorset, comprises a series of stone and flint constructed, and timber-post built, buildings arranged on a courtyard plan, surrounded by a series of ditched enclosures with features associated with industrial use (e.g. kilns/ovens and pits) [20]. The site displays a number of phases of use in Romano-British times between the first and fourth centuries AD. Two mouse samples (OG15 and OG16) were obtained from an extensive deposit of remains of microfauna, which lay on the intact mosaic floor of a room in the main range of buildings, sealed by a deposit of degraded plaster and roof tiles. Analysis of the site and the deposit are ongoing [20], but this appears to represent a deposit of owl pellets, most likely derived from barn owls, which accumulated when the building was going out of use, and was sealed by the collapsed roof. The mosaic floor has been typologically dated to the fourth century AD. Two water vole mandibles from the deposit were successfully subjected to radiocarbon dating (which was possible because this is a relatively large rodent that can yield enough collagen for AMS radiocarbon dating) to elucidate the date of the building collapse, and returned dates of cal. AD 249–391 and cal. AD 208–346 (both 2ơ; [20]).

### Morphological identification

The morphological identification of our 16 mouse mandibles was not easily resolved, as the characters published to distinguish house mouse (*Mus* spp.) from *Apodemus* spp. [21, 22] are not always applicable. Furthermore, some of the archaeological specimens were either missing the M_1_ tooth (OG05 and OG16), where the distinguishing characters are present or visible, or the M_1_ was in an advanced wear stage (OG04, OG05, OG07, OG08 and OG09).

The character we used to determine species identification was the presence of tubercles on the buccal side of the M_1_ in *Apodemus* spp., which are absent in *Mus* spp. [21]. Some of the specimens analysed were suspected to be *Apodemus* spp. but were sampled for DNA to see if the presence of tubercles could be a reliable diagnostic trait (Table 1).

**Table 1:** Details of the ancient murid samples analysed in this study

Specimen	Location	Period	mtDNA Species ID	Total length (bp)	Morphological ID
OG01	Potterne, Wiltshire	LBA/EIA	*Mus musculus domesticus*	74	*Mus* spp.
OG02	Potterne, Wiltshire	LBA/EIA	*Mus musculus domesticus*	772	*Mus* spp.
OG03	Potterne, Wiltshire	LBA/EIA	*Mus musculus domesticus*	576	*Apodemus* spp.?
OG04	Potterne, Wiltshire	LBA/EIA	*Mus musculus domesticus*	772	*Mus* spp./*Apodemus* spp.?
OG05	Potterne, Wiltshire	LBA/EIA	no amplification products	–-	*Mus* spp./*Apodemus* spp.?
OG06	Potterne, Wiltshire	LBA/EIA	*Apodemus sylvaticus*	304	*Apodemus* spp.
OG07	Potterne, Wiltshire	LBA/EIA	*Mus musculus domesticus*	744	*Mus spp./Apodemus spp.?*
OG08	Battlesbury Bowl, Wiltshire	EIA/MIA	*Mus musculus domesticus*	772	*Mus* spp.?
OG09	Battlesbury Bowl, Wiltshire	EIA/MIA	*Mus musculus domesticus*	744	*Mus *spp./*Apodemus *spp.?
OG10	Battlesbury Bowl, Wiltshire	EIA/MIA	*Mus musculus domesticus*	354	*Mus* spp.?
OG11	Battlesbury Bowl, Wiltshire	EIA/MIA	no amplification products	–-	*Mus* spp.?
OG12	Battlesbury Bowl, Wiltshire	EIA/MIA	*Apodemus flavicolis*	259	*Apodemus* spp.
OG13	North West Farm, Dorset	BA?	*Apodemus sylvaticus*	255	*Apodemus* spp.
OG14	North West Farm, Dorset	BA?	No amplification products	–	*Apodemus* spp.
OG15	Druce Farm, Dorset	Roman Period	*Apodemus sylvaticus*	131	*Apodemus* spp.
OG16	Druce Farm, Dorset	Roman Period	*Apodemus sylvaticus*	104	*Mus* spp./*Apodemus* spp.?

*BA?* possible Bronze Age, *LBA* late bronze age, *EIA* EARLY IRON AGE, *MIA* middle iron age

### Extraction and amplification of ancient DNA

We undertook sample processing at the Ancient DNA Facility of the University of Huddersfield (UK) under dedicated clean-room conditions supplied by a positive air pressure system. Researchers wore full body suits, hairnets, gloves and face masks throughout the sampling, extraction and PCR set-up processes, and constantly cleaned all tools and surfaces with LookOut® DNA Erase (SIGMA Life Sciences), as well as with bleach, ethanol and exposures to UV light.

We decontaminated the surface of the mandibulae by UV radiation for 10 min on each side. We shook whole or partial jaws with a zirconium oxide grinding ball inside a zirconium oxide jar in a Mixer Mill (Retsch MM400) for 15 s at 30 Hz/s. We extracted DNA from the resulting 10–50 mg of powder produced following the protocol by Yang et al. [23], with modifications by MacHugh et al*.* [24]. We included blank controls throughout the sampling procedure, extraction, and PCR set-up to monitor for possible contamination.

We amplified and sequenced the mtDNA sequences in 12 overlapping 121–150 bp fragments (Table 2) covering a 915-bp fragment of the control region. We designed four primer pairs specifically for this study, took six from Jones et al*.* [25] with minor modifications, and designed new reverse primers for two primer sets (see Table 2 for more detail). Each primer pair amplified overlapping fragments, including the most variable region between positions 15,381 and 15,663 (when compared to the reference *Mus musculus domesticus* mitochondrial genome, accession number NC_006914).

**Table 2:** Primer pairs used to amplify the ancient mtDNA control-region sequence

Fragment name	Primer name	Primer sequences (5′-3′)	Size (bp)	Reference
Fragment 1	Mm-1F	GCACCCAAAGCTGGTATTCT	146	[25]
	Mm-1R	TTTTATGACCTGAACCATTGATT		Modified from [25]
Fragment 2	Mm-2F	CCAAGCATATAAGCAAGTACAT	141	[25]
	Mm-2R	GTATGTCAGATAACACAGATAT		Modified from [25]
Fragment 2a	Mm-2aF	CAATATATATACCATGAATATTATCTTAA	121	This study
	Mm-2aR	AAGGGGATAGTCATATGG		This study
Fragment 2b	Mm-2bF	ATCTGTGTTATCTGACATACACC	150	This study
	Mm-2bR	TTTAATGGGCCCGGAGCGAGAA		This study
Fragment 2c	Mm-2cF	ACTATCCCCTTCCCCATTTGG	143	This study
	Mm-2cR	GTAAGAACCAGATGTCTGATAA		This study
Fragment 3	Mm-3F	TCTACCATCCTCCGTGAAA	145	Modified from [25]
	Mm-3R	TATGGGCGATAACGCATTTGAT		[25]
Fragment 4	Mm-4F	CTTTATCAGACATCTGGTTCTT	124	[25]
	Mm-4R	CACAGTTATGTTGGTCATGG		This study
Fragment 4b	Mm-4bF	CTTAAATAAGACATCTCGATGG	142	This study
	Mm-4bR	TAGACTGTGTGCTGTCCTT		This study
Fragment 5	Mm-5F	CTTTCATCAACATAGCCGTCAA	129	[25]
	Mm-5R	CATTTATGTCTAACAAGCATGAA		This study
Fragment 6	Mm-6F	CACCTACGGTGAAGAATCATT	146	[25]
	Mm-6R	TGTTTTTGGGGTTTGGCATTAA		[25]
Fragment 7	Mm-7F	CTCAATACCAAATTTTAACTCTC	144	[25]
	Mm-7R	GTCATATTTTGGGAACTACTAG		[25]
Fragment 8	Mm-8F	CTATCAAACCCTATGTCCTGA	140	[25]
	Mm-8R	CTTGTTAATGTTTATTGCGTAA		Modified from [25]

We designed three of the pairs of primers (2b, 2c and 3) that can also amplify the DNA of *Apodemus*. This was done to help identify mandibulae that presented identification difficulties where identification as Mus was uncertain.

### Analyses of ancient and modern house mouse data

Phylogenetic reconstruction from *Mus* control-region data is challenging, generating even more homoplasy than is seen in modern humans. We estimated phylogenies in two different ways. We firstly aligned the eight ancient sequences (OG01, OG02, OG03, OG04, OG07, OG08, OG09 and OG10) obtained for *Mus musculus domesticus* in this study with 728 previously published house mouse sequences from Austria, Bulgaria, Great Britain, Croatia, Denmark, France, Germany, Greece, Italy, Ireland, Norway, Portugal, Spain and Sweden (see Additional File 1: Table S2) [7, 25–28, 31, 33–36] to create a Bayesian inference phylogenetic tree with MrBayes [29], using the parameters previously calculated in JModelTest [30]. We ran the analysis for five million generations with four chains and with a 25% burn-in. We used FigTree v.1.3.1 to visualise the tree, and haplogroups were assigned and named following previous nomenclature [26].

The necessity of using shorter sequence lengths in the aDNA analysis meant that it is not possible to identify all of the clades revealed in the original published house mouse analyses (that used longer sequence lengths). Therefore, we used these data to estimate haplogroup phylogenies using the Network software (see Additional File 1: Table S3), constructing separate networks for lineages from haplogroups D and E using Network v.5.0.1.1 (www.fluxus-engineering.com). After an initial run to separate the major haplogroups (not shown), we first made the data binary where necessary (at positions with both a transition and a transversion, or a transition and a deletion), and then ran the reduced-median algorithm, followed by the median-joining algorithm, on the pre-processed dataset. We included indels, as a proportion of the variation in the mouse control region comprises insertion events; however, where there were tracts of contiguous indels (such as the 11 base pair insertion seen in some D1 individuals between positions 16,089 and 16,090 when compared to the reference mitogenome, NC_006914), we only counted these as a single event. We estimated the position of the root of each haplogroup network from the larger network.

## Results

### Sample identification

In total, we obtained mtDNA sequence data from 13 of the 16 samples, of which we identified eight individuals as *Mus musculus domesticus*, four as *Apodemus sylvaticus*, and one as *Apodemus flavicolis* (Table 2). The Bronze Age North West Farm site only yielded DNA from one individual, attributed to *A. sylvaticus*, and the Roman period Druce Farm site also yielded only *A. sylvaticus* (*n* = 2). Other *Apodemus* spp. samples were found at the Late Bronze Age/Early Iron Age site of Potterne (*A. sylvaticus*; *n* = 1) and the Iron Age Battlesbury site (*A. flavicolis*; *n* = 1). However, in both of these sites, eight *M. m. domesticus* samples were also found (five at Potterne and three at Battlesbury). These results highlight the uncertainty in the identification of murid species in the archaeological record based on mandible morphology, particularly if the M_1_ is either worn or absent. We tested the reliability of the presence of tubercles on the buccal side of the M_1_ being a diagnostic trait for *Apodemus* spp., as these are absent in *Mus* spp. [21]. In one case, OG03, the M_1_ was present, and unworn tubercles appeared also to be present, suggesting that the specimen belonged to *Apodemus.* However, the specimen was partially obscured by sediment, and the mtDNA indicated the specimen to be *Mus* spp. (Table 2). In all other cases, the diagnostic nature of the tubercles proved reliable, and aDNA was shown to be a useful tool for species identification if wear or a lack of M_1_ did not allow the trait to be used.

### House mouse phylogeography in Britain

**Fig. 1:**
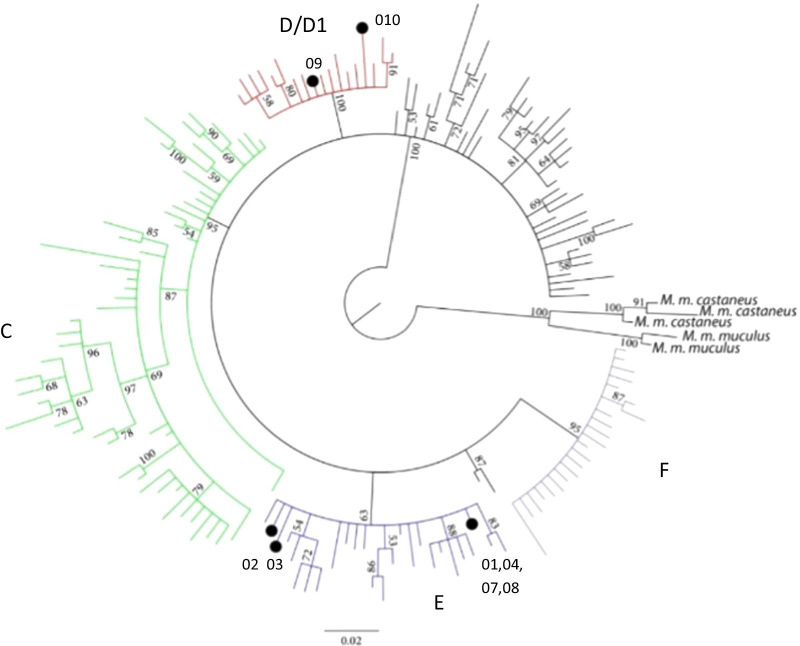
Bayesian phylogenetic tree constructed from 772 bp of house mouse control region sequences (from positions 15,508 to 16,279 of the reference house mouse mitogenome, NC_006914). The tree includes 728 modern and eight ancient British sequences (Additional File 1: Table S2). Ancient samples are indicated with a black dot. Bootstrap values of greater than 50 are indicated on the branches

The longest fragment obtained from *M. m. domesticus* was 772 bp (OG04), from positions 15,508 to 16,279 of the reference house mouse mitogenome (NC_006914; Table 2). The shortest fragment (OG01) had a length of only 74 bp. All house mouse individuals analysed here date to the Late Bronze Age or the Iron Age, and they clustered within two main haplogroups (D and E; Table 3; Fig. 1), described previously in modern samples from Britain.

**Table 3:**
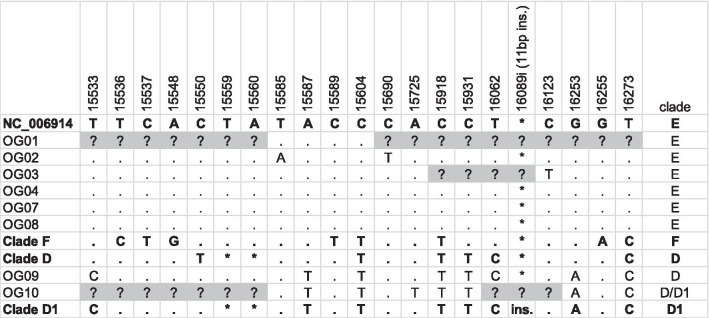
Variable positions in control region sequences of archaeological *Mus musculus domesticus* samples from the British Isles between positions 15,508 and 16,279, compared with the reference house mouse mitogenome (NC_006914; haplogroup E), and with reference sequences from haplogroups D, D1 and F

Differences in bases are indicated in the table for each sample, a full stop denotes identical bases and an asterisk denotes a deletion. Missing sequence data from the ancient samples are denoted by question marks. Haplotype within clade D1 have an 11 bp insertion at position 16089i, which is a repeat of the sequence seen between 16,079 and 16,089 (TTTTAACTCTC). Sequence codes are given in the first column. In the final column, each sample has been assigned to a mitochondrial clade by means of its relative position in the larger network, and the variant sites compared with modern samples

Six individuals (from across both sites) belonged to haplogroup E and two more (from Battlesbury Bowl) clustered in haplogroup D. Haplogroup F, the most widespread cluster in Britain today, was not present in our sample set. Three samples (OG04, OG07 and OG08) belonged to the same haplotype within E.

**Fig. 2:**
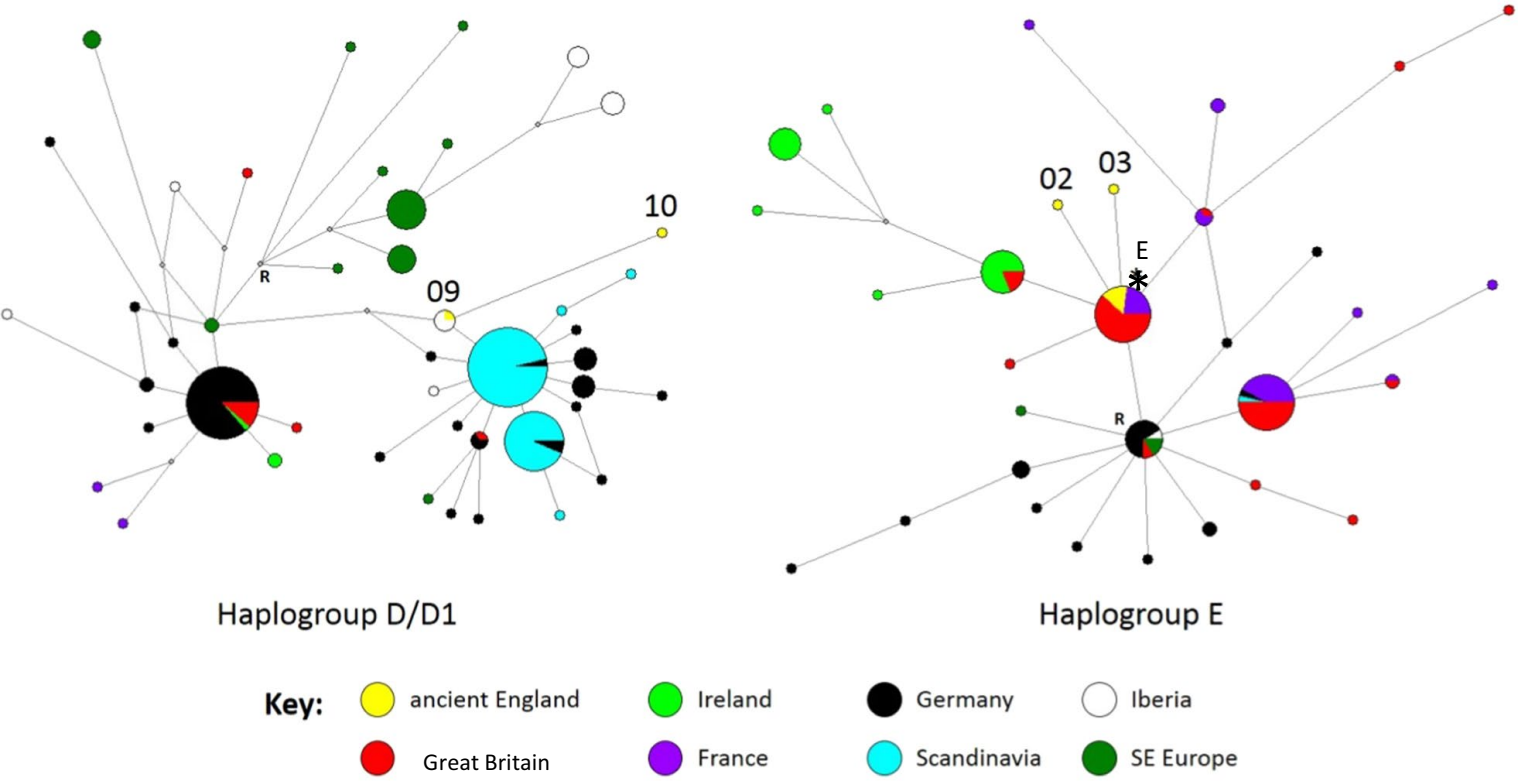
Phylogenetic networks for haplogroups D and E, constructed from 772 bp of house mouse control region sequences (from positions 15,508 to 16,279 of the reference house mouse mitogenome, NC_006914). The modern sequences used to construct each network are shown in Additional File 1: Table S3. Haplogroup D network includes two of the ancient house mouse sequences (both from Battlesbury Bowl), while the haplogroup E network includes six of the ancient house mouse sequences (five Potterne and one Battlesbury Bowl). The ancient samples include OG01, 03, 09 and 10, with the asterisk in the haplogroup E network indicating OG02, 04, 07 and 08. Where there are missing data in the ancient sequences (Table 3), the sample has been placed determined on its most likely position based on the phylogenetic network. The precise position of OG10 within the D/D1 clade in the phylogenetic network may be unreliable due to missing data. Circles represent sequence haplotypes, the area being proportional to the frequency, and they are coloured by the location of the samples, as seen in the accompanying key. Small grey points are reconstructed intermediate nodes introduced by the network algorithm, and links between haplotypes represent mutations. R denotes the likely position of the root of each network, as determined from the full network (not shown)

The haplogroup networks (Fig. 2) locate the ancient British samples within haplogroups D and E. All five Potterne samples and the Battlesbury Bowl haplogroup E sequence are securely located in close proximity to French/British/Irish samples within haplogroup E. Four of them directly match the root haplogroup of this group, found both in modern Britain and France, which is related to lineages seen in Potterne, modern Great Britain and Ireland. The two remaining Battlesbury Bowl samples fall within haplogroup D. Haplogroup D is itself rare in present-day Britain and confined to the north in current datasets [26, 31].

Unfortunately, the fragments obtained for the *Apodemus* spp., whilst distinctive, were too short for meaningful integration into wood mouse (*A. sylvaticus*) or yellow-necked mouse (*A. flavicolis*) phylogeographies.

## Discussion

**Fig. 3:**
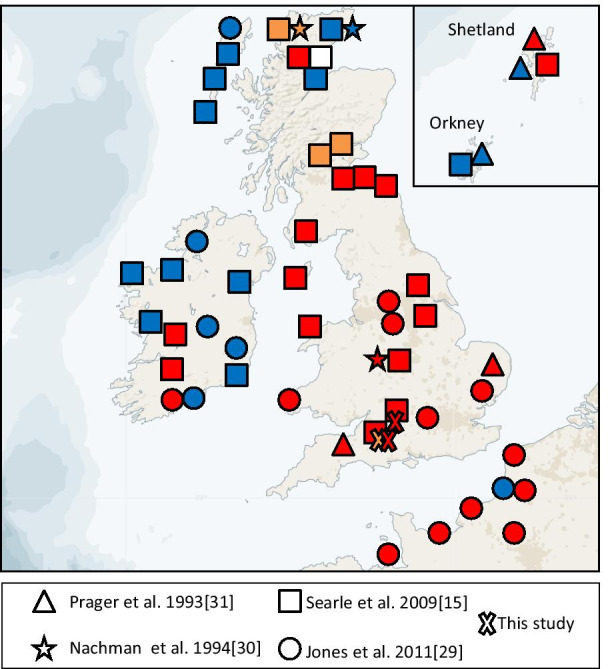
Localities and mtDNA haplogroups for all samples of *Mus musculus domesticus*, the western European house mouse, from the British Isles and northern France. Colours indicate the control region haplogroup presence with several individuals (white: haplogroup C; orange: haplogroup D; red: haplogroup E; blue: haplogroup F). Branches are not proportional to the number of mutations. Circles, squares, stars and triangles show previously published sequence data as denoted in the key. Crosses show the localities where ancient DNA has been recovered as part of this study. Credit: Image adapted from Mapbox (www.mapbox.com)

If *M. m. domesticus* started its spread from the Near East, associated with modern humans, during the Early Neolithic [3, 37, 38], then Britain is at the periphery of the western European expansion of the subspecies. The phylogeography of the western part of the Atlantic geographical range of the house mouse has been particularly well studied [7, 26, 39] and, based on haplotype diversity and the presence of different clades, Britain has a relatively high genetic variation [7]. This study complements the understanding of the colonisation of the British Isles by the western European house mouse by showing that two haplogroups, D and E, were present in southern Great Britain by the time of the Iron Age, although haplogroup E may have arrived earlier based on its presence on the Late Bronze Age/Early Iron Age archaeological site (Fig. 3). These two clades may represent several different mouse migrations linked to different human movements.

Haplogroup E is the most common *M. m. domesticus* lineage in modern southern and central British samples and is also present in Scotland and southern Ireland (Figs. 1, 2 and 3) [7, 26]. Searle et al. [7] suggested that the distribution of haplogroup E might reflect the colonisation of Great Britain from the European mainland during the Iron Age. As this clade is not well represented in central Europe, it has been suggested that they did not arrive with people overland but via a maritime route, possibly transported from the Mediterranean by the seafaring Phoenicians in the Late Bronze Age/Early Iron Age [32]. The network of E, even with the truncated sequences, (Fig. 2) could point toward a putative proximal source in Germany for the French, British and Irish lineages, with several distinct subclades, most of which Great Britain shares with France, and a suggestion of a possible ancestry further back in Portugal, and ultimately Greece, as previously published [32]. Although zooarchaeological evidence has been used to argue that there was little or no house mouse presence in western Europe until the urban developments of the Iron Age [3], our results complement the possibility that the extant haplogroup E lineages may have reached southern Great Britain as early as the Late Bronze Age.

Haplogroup D is much less common in Britain today (Fig. 3). It is distributed at low frequencies from the Levant to the central and western Mediterranean, and north into Germany, northern Britain and the Baltic. Its derived subclade, D1, co-occurs with the remainder of D in Germany, but has replaced it in Denmark and Sweden (but not Norway), where D1 reaches 100%. D1 is also seen in Madeira and the Canary Islands [31, 33, 39], and the colonisation of these islands has been attributed to Danish Viking movements, first to Madeira and then to the Canary Islands, following Portuguese settlement [31, 39]. Only a single instance of D1 has been recorded in the British Isles, from the far north, and haplogroup D overall is restricted to Scotland in current published data.

The presence of haplogroup D in Britain in the Iron Age, and more particularly in southern England, may suggest an introduction from continental Europe—either Germany or Denmark, or possibly even Iberia. These lineages are phylogenetically distinct from extant British D lineages, which appear more directly linked to lineages from Germany, and may be unconnected to them historically (Fig. 2). Haplogroup D may, therefore, have been introduced to Britain on at least two distinct occasions, all of them possibly separate from the introduction of haplogroup E (Fig. 3).

Although three main haplogroups are represented in modern British samples (D, E and F, with F being the most frequent; Fig. 3), only two of these (D and E) have been found in the archaeological sites analysed here. The presence of haplogroups D and E in Britain since at least the Iron Age had previously been hypothesised from the analysis of modern samples [25, 26, 32]. This study provides the first direct evidence of the presence of D and E in Britain, and specifically southern England, from at least the Iron Age period, and the lack of haplogroup F in our data is consistent with its later introduction, possibly associated with the Vikings [7].

## Conclusion

The presence of haplogroups D and E during the Late Bronze Age and Iron Age in Great Britain has provided evidence of an early house mouse colonisation that may be related with first-millennium expansions of humans. This is broadly in agreement with what has been previously suggested on the basis of modern mtDNA data, although the presence of haplogroup E possibly in the Late Bronze Age would predate, by several centuries, the most widely accepted model for its expansion in Europe [32, 38].

## Supplementary Information

**Additional file 1:** Supplementary material including Table S1, Table S2, Table S3 and Figure S1.

## Data Availability

All the ancient British house mouse mtDNA sequences generated in the course of the study have been deposited in GenBank (Accession Numbers MW459973-MW459980). Public access to the database is open.
