# Supplementary material for: Ancient mitochondrial DNA connects house mice in the British Isles to trade across Europe over three millennia
Source: BMC Ecol Evol. 2021 Jan 23;21:9. doi: 10.1186/s12862-021-01746-4 (PMC7853306; doi:10.1186/s12862-021-01746-4)
Supplement: Supplementary file 1 — Additional file 1: Supplementary material including Table S1, Table S2, Table S3 and Figure S1. [file 12862_2021_1746_MOESM1_ESM.docx]

**SUPPLEMENTARY MATERIAL**

**Table S1.** Table of archaeological *Mus/Apodemus* specimens studied, with associated period and context information.

| **Specimen** | **Location** | **Period** | **Context information** |
| --- | --- | --- | --- |
| OG01 | Potterne, Wiltshire | Late Bronze Age/Iron Age | N35 134 122 Cutting 12 |
| OG02 | Potterne, Wiltshire | Late Bronze Age/Iron Age | W35 221 5 159 Cutting 12 |
| OG03 | Potterne, Wiltshire | Late Bronze Age/Iron Age | W35 202 565 Cutting 12 |
| OG04 | Potterne, Wiltshire | Late Bronze Age/Iron Age | W35 202 564 Cutting 12 |
| OG05 | Potterne, Wiltshire | Late Bronze Age/Iron Age | W35 221 5160 Cutting 12 |
| OG06 | Potterne, Wiltshire | Late Bronze Age/Iron Age | W35 3716 5.676R Cutting 12 |
| OG07 | Potterne, Wiltshire | Late Bronze Age/Iron Age | W35 221 5160 Cutting 12 |
| OG08 | Battlesbury Bowl, Wiltshire | Early/Middle Iron Age | W4896 4817 <2143> |
| OG09 | Battlesbury Bowl, Wiltshire | Early/Middle Iron Age | W4896 4817 <2143> |
| OG10 | Battlesbury Bowl, Wiltshire | Early/Middle Iron Age | W4896 4174 <2010> |
| OG11 | Battlesbury Bowl, Wiltshire | Early/Middle Iron Age | W4896 5137 |
| OG12 | Battlesbury Bowl, Wiltshire | Early/Middle Iron Age | W4896 5056 |
| OG13 | North West Farm, Dorset | Bronze Age | NWF17 340 "2" bag 79 |
| OG14 | North West Farm, Dorset | Bronze Age | NWF17 340 "1" bag 78 |
| OG15 | Druce Farm, Dorset | Roman Period | DF13 (2) (197) HXAM |
| OG16 | Druce Farm, Dorset | Roman Period | DF13 (2) (197) HXAM |

**Table S2**. Details of the 728 previously published house mouse sequences used in the Bayesian tree (**Figure 1**).

| **Location** | **#** | **Reference(s)** |
| --- | --- | --- |
| Austria | 1 | Prager et al. (1996) |
| Bulgaria | 24 | Rajabi-Mahamet al. (2008) |
| Britain | 97 | Prager et al. (1993); Nachman et al. (1994); Searle et al. (2009); Bonhomme et al. (2011) |
| Croatia | 3 | Prager et al. (1993); Prager et al. (1996) |
| Denmark | 114 | Prager et al. (1993) |
| England | 10 | Jones et al. (2011); Jones et al. (2012) |
| France | 94 | Ihle et al. (2006); Bonhomme et al. (2011); Jones et al. (2011); Jones et al. (2012) |
| Germany | 112 | Prager et al. (1993); Ihle et al. (2006); Bonhomme et al. (2011) |
| Greece | 8 | Prager et al. (1993); Nachman et al. (1994) |
| Italy | 62 | Prager et al. (1993); Nachman et al. (1994); Rajabi-Mahamet al. (2008) |
| Ireland | 69 | Jones et al. (2011); Jones et al. (2012) |
| Norway | 9 | Prager et al. (1996); Searle et al. (2009) |
| Portugal | 77 | Prager et al. (1993); Gündüz et al. (2001) |
| Scotland | 6 | Jones et al. (2011); Jones et al. (2012) |
| Spain | 11 | Prager et al. (1993); Nachman et al. (1994); Bonhomme et al. (2011) |
| Sweden | 30 | Prager et al. (1993) |
| Wales | 1 | Jones et al. (2011); Jones et al. (2012) |

**Table S3.** Details of published modern European house mouse sequences used for constructing phylogenetic networks for haplogroups D and E (**Figure 2**).

| **Country** | **# in D network** | **# in E network** | **Reference(s)** |
| --- | --- | --- | --- |
| Britain | 8 | 29 | Prager et al. (1993); Nachman et al. (1994); Bonhomme et al. (2011) |
| England | 0 | 10 | Jones et al. (2011); Jones et al. (2012) |
| Wales | 0 | 1 | Jones et al. (2011); Jones et al. (2012) |
| Ireland | 3 | 25 | Jones et al. (2011); Jones et al. (2012) |
| France | 2 | 25 | Ihle et al. (2006); Bonhomme et al. (2011); Jones et al. (2011) |
| Germany | 69 | 21 | Prager et al. (1993); Ihle et al. (2006); Bonhomme et al. (2011) |
| Denmark | 54 | 0 | Prager et al. (1993) |
| Norway | 0 | 1 | Prager et al. (1996); Searle et al. (2009) |
| Sweden | 30 | 0 | Prager et al. (1993) |
| Portugal | 9 | 1 | Prager et al. (1993); Gündüz et al. (2001) |
| Spain | 6 | 0 | Prager et al. (1993); Nachman et al. (1994); Bonhomme et al. (2011) |
| Bulgaria | 3 | 0 | Rajabi-Mahamet al. (2008) |
| Greece | 0 | 3 | Prager et al. (1993); Nachman et al. (1994) |
| Italy | 27 | 0 | Prager et al. (1993); Nachman et al. (1994); Rajabi-Mahamet al. (2008) |


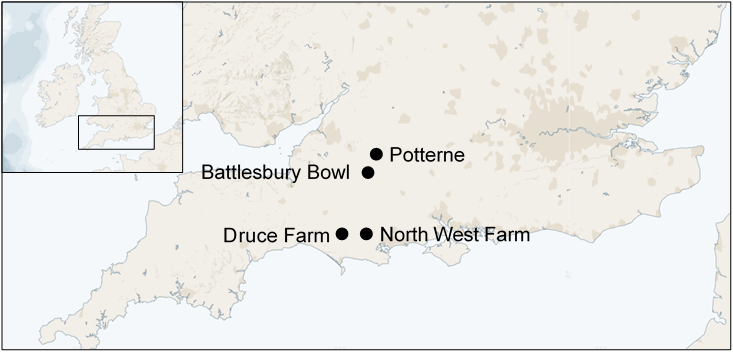


**Figure S1.** Map of the archaeological sites sampled in this study. Image adapted from Mapbox (www.mapbox.com)
